# Long-Term Experience of Arterio-Venous Fistula Surgery in Children on Hemodialysis

**DOI:** 10.3390/jcm13123577

**Published:** 2024-06-18

**Authors:** Veronika Almási-Sperling, Christine Gall, Briain Haney, Nina Latzel, Ferdinand Knieling, Alina C. Hilger, Adrian P. Regensburger, Alexander Meyer, Werner Lang, Ulrich Rother

**Affiliations:** 1 Department of Vascular Surgery, University Hospital Erlangen, Friedrich-Alexander-Universität Erlangen-Nürnberg (FAU), Krankenhausstraße 12, 91054 Erlangen, Germany; veronika.almasi@uk-erlangen.de (V.A.-S.); briain.haney@uk-erlangen.de (B.H.); nina.latzel@ksa.ch (N.L.); werner.lang@uk-erlangen.de (W.L.); 2 Department of Medical Informatics, Biometry and Epidemiology, University of Erlangen-Nuremberg, 91054 Erlangen, Germany; 3 Department of Pediatrics and Adolescent Medicine, University Hospital Erlangen, Friedrich-Alexander-Universität Erlangen-Nürnberg (FAU), Loschgestraße 15, 91054 Erlangen, Germany; ferdinand.knieling@fau.de (F.K.); alina.hilger@uk-erlangen.de (A.C.H.); adrian.regensburger@uk-erlangen.de (A.P.R.); 4 Department of Vascular Surgery, Helios Klinikum Berlin-Buch, Schwanebecker Chaussee 50, 13125 Berlin, Germany; alexander.meyer3@helios-gesundheit.de; 5 Medical School Berlin, 14197 Berlin, Germany

**Keywords:** arterio-venous fistula, children, primary patency, secondary patency, long term

## Abstract

**Background**: Arterio-venous fistulas (AVF) are used as first-line access for hemodialysis (HD) in the pediatric population. The aim of this investigation was to describe a single-center experience in the creation of AVF, together with its patency in children. **Methods**: This single-center retrospective study included all patients aged ≤18 years with AVFs created between 1993 and 2023. The collected data included patients’ demographics, hemodialysis history, intraoperative data, and required reinterventions in order to determine the impact of these variables on primary, primary-assisted, and secondary patency. **Results**: Fifty-seven patients were analyzed with a median age of 15 years (range, 7–18 years). Fifty-four forearm and four upper arm fistulas were performed. The median follow-up was 6.9 years (range, 0–23 years). The primary failure rate was 10.5%. The primary patency rate was 67.6%, 53.6%, 51.4%, and 38.1% after 1, 3, 5, and 10 years; primary-assisted patency was 72.9%, 62.8%, 60.6%, and 41.5%; and secondary patency was 87.3%, 81.3%, 76.8%, and 66.6% after 1, 3, 5, and 10 years in the studied population. **Conclusions**: AVFs showed an acceptable rate of primary failure and excellent long-term patency. In this context, AVFs are an appropriate option for HD access, especially in pediatric patients.

## 1. Introduction

Chronic kidney disease (CKD) is a progressive and often multifactorial disease in older patients with pre-existing conditions such as high blood pressure and diabetes [1]. While 13.4% of the global population already has CKD [2], the number of patients per year requiring renal replacement therapy is increasing. Despite this increasing number, mainly associated with increasing life expectancy and the prevalence of type 2 diabetes (T2DM), obesity, hypertension, and cardiovascular diseases [3,4,5], the causes in children may be different. Although this concept is similar to adults, the origin of CKD is most often associated with congenital anomalies of the kidney and urinary tract (CAKUT) [6]. In cases in which CKD is proceeding to its end stage, preemptive kidney transplantation currently remains the recommended therapy in pediatric patients [7].

While awaiting kidney transplantation, the primary choices of vascular access for HD are arterio-venous fistulas (AVFs), arterio-venous grafts (AVGs), or central venous catheters (CVCs) [8]. The National Kidney Foundation Dialysis Outcome Quality Initiative (K/DOQI) recommends the use of AVF as the vascular access of choice [9] for HD; however, in daily practice, there is still a tendency to favor CVC for short-term dialysis, often resulting in increased morbidity during long-term use [10]. Advantages of AVF in comparison to CVC include lower rates of infection and thrombosis, the longevity of the access site, high dialysis adequacy, and a higher freedom for daily activities. Benefits of CVC include needle-free dialysis, immediate delay-free access, greater freedom of movement during dialysis, and immediate usability after placement [11]. Currently, CVCs remain the most commonly used form of access for chronic HD in children, with a rate of 73–87.6% vs. 11–26% for AVF [12,13,14].

While the feasibility of AVF creation in children has been described earlier [12,15,16,17], only a few studies have examined the long-term outcomes in this population [18]. In consequence, there are insufficient data examining risk factors that have an impact on long-term patency [19]. The purpose of this study was to examine predictors that influence the maturation and long-term patency of AVF after creation in children based on 30 years of experience.

## 2. Materials and Methods

### 2.1. Patients

A retrospective, single-center analysis of consecutive pediatric patients treated for AVF creation at a university hospital was conducted. Data of patients who met the inclusion criteria and were operated on between February 1993 and February 2023 were retrospectively analyzed. The study was conducted in congruence with the declaration of Helsinki, adhered to the STROBE (Strengthening the Reporting of Observational Studies in Epidemiology) guidelines [11], and was further approved by the local ethics committee (23–369-Br). Written informed consent was not necessary, given the retrospective character of the study.

### 2.2. Inclusion and Exclusion Criteria

Consecutive patients (age ≤ 18 years) treated for AVF creation were included. Preemptive CKD stage 5 (eGFR less than 15 mL/min) patients and patients with metabolic disorders (methylmalonic acidemia or hyperlipidemia) were included. There were no restrictions concerning the causes of kidney failure for this type of fistula. In consequence, AVF, as well as alternative arterio-venous graft (AVG), were included. All patients were in need of long-term dialysis, as they were either unsuitable for kidney transplantation or had a waiting time for transplantation of over 6 months.

### 2.3. Preoperative Evaluation and Operative Technique

**Figure 1:**
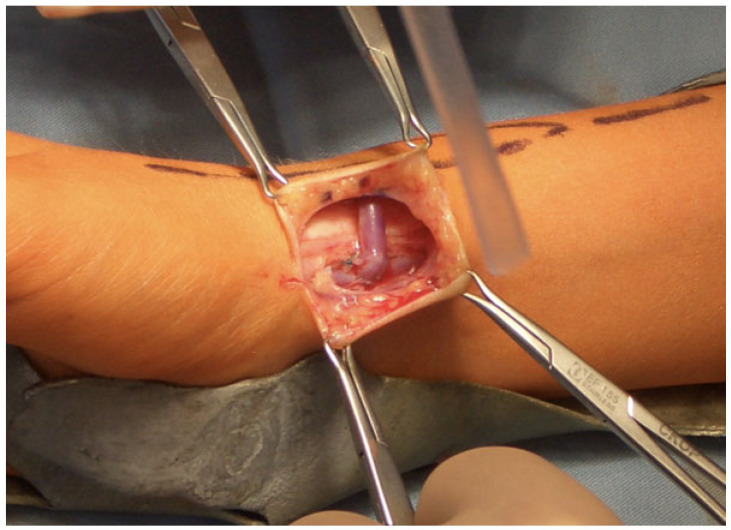
Clinical image of an arterio-venous fistula in a 6-year-old boy.

All patients screened for AVF creation underwent a standard evaluation procedure. Preoperative vein mapping for vessel evaluation via ultrasound was performed. Native vessels were used if the vein and artery diameter was at least 1.5 mm. In addition, the patency and continuity of the vessels were assessed using duplex ultrasound. The creation of each fistula was performed under inpatient conditions and general anesthesia exclusively by vascular surgeons with more than 5 years of professional experience using microsurgery techniques. All AVFs were created using a loupe magnification with a standard end-to-side anastomosis and a monofilament suture in an interrupted or continuous technique, depending on fistula location (Figure 1). In order to decrease the risk of clamping-induced dissection or vasospasm, tourniquet occlusion was used for inflow control in selected cases. Papaveron^®^ (Papaverine, Tropitzsch GmbH, Marktredwitz, Germany) was used during surgery to reduce vasospasm. The application of intraoperative anticoagulation was based on the decision of the vascular surgeon and depended on vessel diameter as well as the occurrence of vasospasms. There was no routine use of anticoagulation or platelet aggregation inhibitors after access creation in the postoperative period. 

### 2.4. Definitions and Data Collection

Patient parameters, including demographics, hemodialysis history, intraoperative data, patency rates, and long-term outcomes, were retrospectively collected. Patient files were assessed, and local hemodialysis centers, as well as the patients’ general practitioners, were contacted for further information in order to ensure clean data collection. Primary failure was defined as the inability to use the fistula even once due to insufficient maturation or occlusion within 14 days after creation. Primary patency was defined as the interval from the time of access creation to the development of the first problems or reestablished patency by revision. Primary-assisted patency represents the interval between fistula creation and the first occlusion. Secondary patency was defined as the total lifespan from creation to permanent access failure. The observation time was censored if the AVF was given up because it was no longer needed, e.g., due to kidney transplantation or if lost to follow-up or by the end of the observation period.

Primary failure, patency rates, and long-term outcomes were recorded for a median follow-up of 6.9 years (range, 0–23 years). Special predictors and their influence on maturation were analyzed. We compared groups with a special focus on the duration of preoperative dialysis. We defined groups of patients with hemodialysis >1 year before fistula creation (Group A), <1 year before fistula creation (Group B), and hemodialysis after fistula creation (Group C). Other predictors like age, body weight, and fistula localization were also included in the analysis.

### 2.5. Statistical Analysis

The statistical analysis was carried out using the R software (Version 4.2.2). Event times were calculated from the time of AVF surgery. In the case of patients needing a second AVF, the follow-up was terminated by the date of the second AVF surgery. The Kaplan–Meier method was used to estimate event probabilities. The log-rank test was used for comparison.

## 3. Results

### 3.1. Patient Characteristics

In total, 57 children (29 male, 28 female) were included. The median age at the time of the operation was 15 years (range, 7–18 years), and the median weight was 47 kg (range, 19–81 kg). Etiologies of renal failure were glomerulonephritis (*n* = 25), obstructive uropathy (*n* = 23), metabolic diseases (*n* = 3), metacystic dysplastic kidney (*n* = 2), or unknown (*n* = 2). Two patients received AVF without renal failure in order to perform lipid apheresis due to hyperlipidemia. During the study period, 57 AVFs were created. At the time of surgery, 24 patients were already on dialysis; of those, 20 patients were undergoing HD through a CVC, and 4 patients underwent peritoneal dialysis (Table 1).

### 3.2. AVF Characteristics

In total, 54 forearm and 3 upper arm fistula operations were performed. A Cimino-Brescia fistula was performed in 34 cases on the forearm. Other forearm fistulas were created between the basilic vein and the radial artery or using the cephalic vein of the forearm in a loop technique. Arterio-venous grafts with prosthetic material were not performed in any of the patients. A detailed overview of the different locations and configurations is given in Table 1.

### 3.3. Patency Rates and Long-Term Outcome

**Figure 2:**
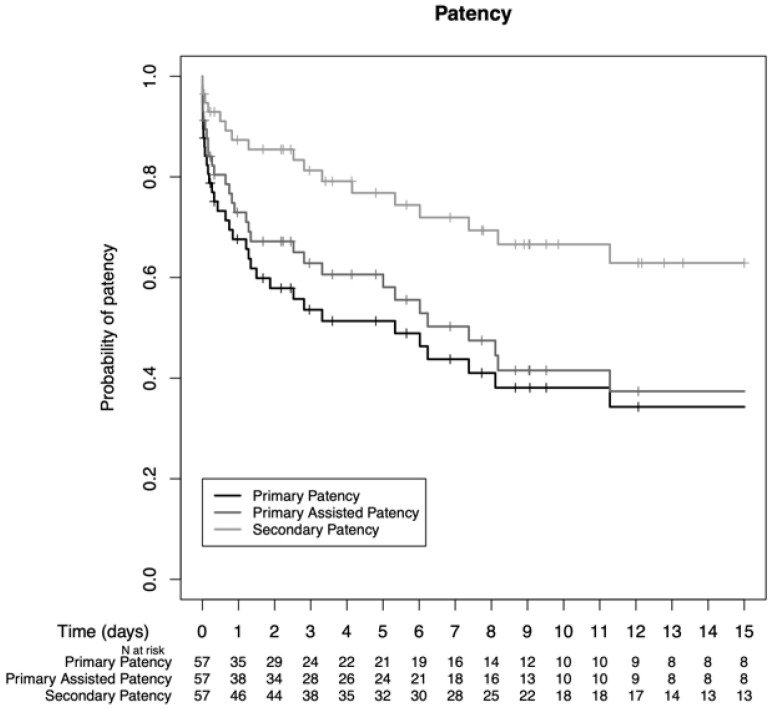
Primary, primary-assisted, and secondary patency rates in the study period.

The median follow-up duration was 6.9 years (range, 0–23 years). During the entire study period, five patients were lost to follow-up. Primary patency rates were 67.6%, 53.6%, 51.4%, and 38.1% after 1, 3, 5, and 10 years. Primary-assisted patency rates were 72.9%, 62.8%, 60.6%, and 41.5%; secondary patency rates were 87.3%, 81.3%, 76.8%, and 66.6% after 1, 3, 5, and 10 years in our pediatric population, respectively (Figure 2).

**Figure 3:**
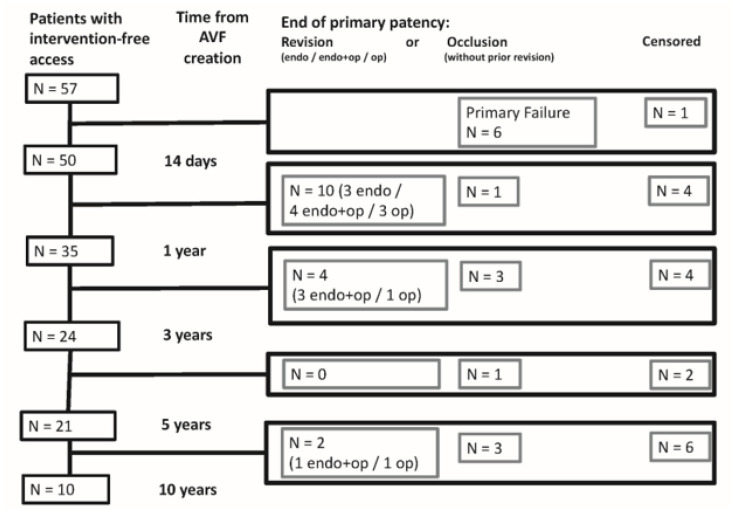
Study flow chart showing the primary patency over a 10-year period; additionally, the reasons for the end of primary patency are given. The revision procedure was categorized as endo (endovascular procedure), op (surgical revision), or endo + op (endovascular procedure followed by surgical revision).

The primary failure rate in the first 2 weeks after fistula creation was 10.5% (*n* = 6). For maintaining the fistula patency in 22 of the 57 patients, interventions were necessary. In the patients needing re-interventions, an average of 2.3 procedures (range 1–7, either endovascular or open-surgical) were conducted. Figure 3 shows the interventions needed, leading to the end of primary patency.

Of the 57 AVFs initially created, 46 AVFs were cannulated and used for hemodialysis. The remaining 11 AVFs were never cannulated in the study period due to coinciding kidney transplantation. Kidney transplantation status was also recorded. Thirty-two patients (56%) received a kidney transplantation during the study period. The mean waiting time for transplantation was 8.7 years. Three patients underwent kidney transplantation before AVF creation.

### 3.4. Factors Influencing Patency Rates

Different factors were assessed to determine their influence on patency rates. However, none of the investigated factors had a significant influence.

#### 3.4.1. Age

**Figure 4:**
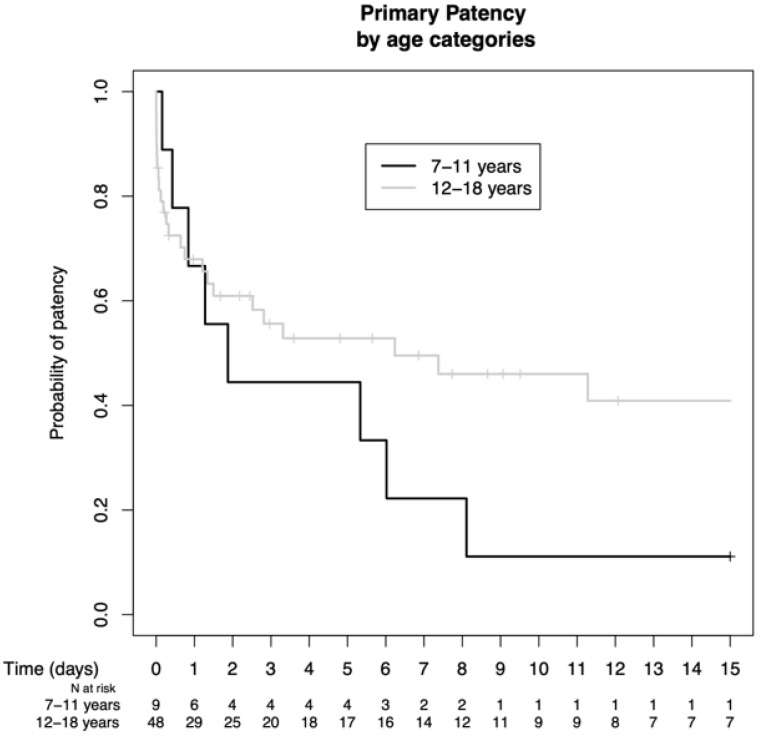
Comparison of the primary patency rates according to different age categories.

The patients were grouped as 7–11 years and 12–18 years old, and the patency rates in the according groups were assessed. However, there was no statistically significant increase in primary patency in older patients (66.7% vs. 68.0% at 1 year, 44.4% vs. 55.6% at 3 years, 44.4% vs. 52.8% at 5 years, and 11.1% vs. 46.0% at 10 years, *p* = n.s.) (Figure 4).

#### 3.4.2. Body Weight

**Figure 5:**
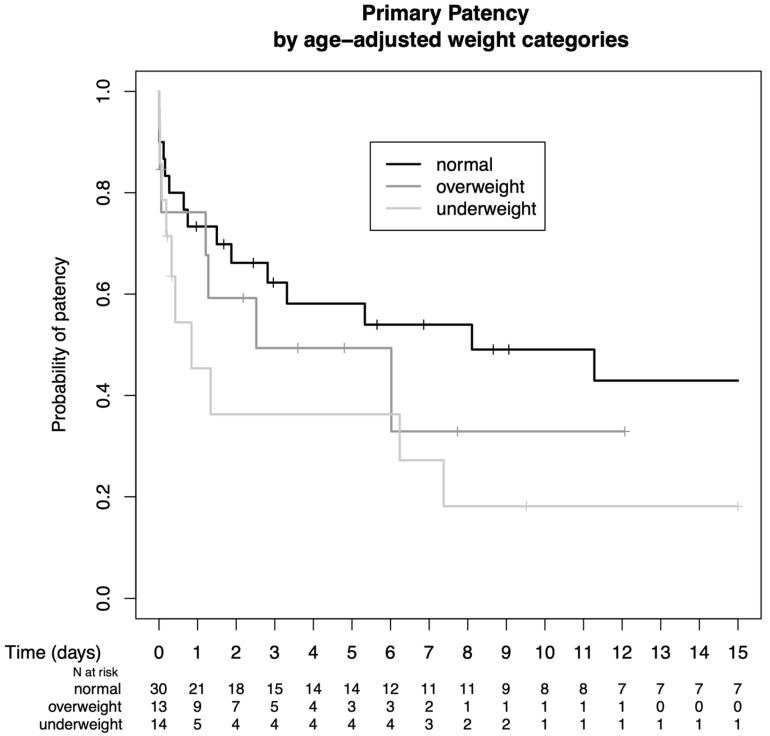
Comparison of primary patency rates according to age-adjusted weight categories.

Patients were grouped by age-adjusted weight categories as underweight, normal weight, or overweight. Normal weight seemed to yield a benefit in terms of the durability of the AVF; however, this was not statistically significant. (Primary patency rates: 45.4% vs. 73.3% vs. 76.2% at 1 year, 36.3% vs. 62.3% vs. 49.4% at 3 years, 36.3% vs. 58.1% vs. 49.4% at 5 years, and 18.1% vs. 49.1% vs. 32.9% at 10 years, *p* = n.s.) (Figure 5).

#### 3.4.3. Localization

The different locations of the AVFs were compared concerning their primary patency rates (Cimino fistulas vs. other AVF types in the forearm as well as in the upper arm). The primary patency rates were 64.7% vs. 71.7% at 1 year, 49.3% vs. 60.7% at 3 years, 45.5% vs. 60.7% at 5 years, and 25.0% vs. 60.7% at 10 years (*p* = n.s.).

#### 3.4.4. Hemodialysis before Fistula Creation

The study cohort was further divided according to the duration of preoperative dialysis. Group A (*n* = 4) included patients with hemodialysis >1 year before fistula creation, Group B (*n* = 13) <1 year before fistula creation, and Group C (*n* = 40) with no hemodialysis before fistula creation. There was no tendency for increased patency rates in any group (75% vs. 53.8% vs. 71.8% at 1 year, 37.5% vs. 46.2% vs. 57.5% at 3 years, 37.5% vs. 46.2% vs. 54.1% at 5 years, and 37.5% vs. 27.7% vs. 41.7% at 10 years, *p* = n.s.).

#### 3.4.5. Cannulation

**Figure 6:**
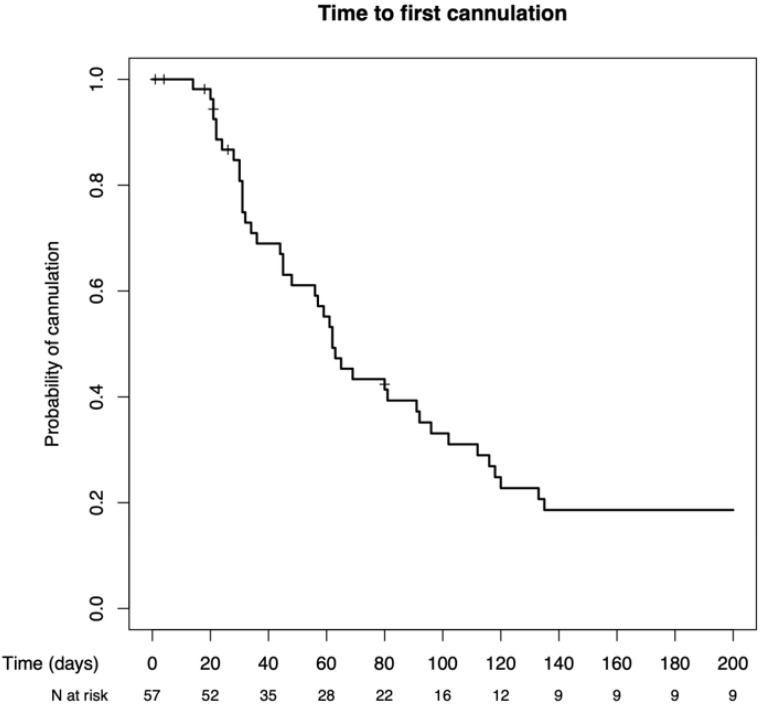
Time to first cannulation of the fistulas cannulated.

The time of the first cannulation depended on the decision of the pediatric nephrologist and varied widely among the study population (Figure 6). Consequently, the time point of the first fistula cannulation did not show any influence on the primary patency rates (*p* = n.s.).

## 4. Discussion

In this single-center retrospective study of 57 patients, we observed an acceptable rate of primary failure and excellent long-term patency of AVFs. Children with CKD developing irreversible kidney function damage frequently progress to end-stage renal disease requiring dialysis. Although relevant epidemiological evidence indicates that CKD is a major and widely growing public health crisis in adults [20], limited data are available regarding the epidemiology of pediatric CKD. According to the United States Renal Data System (USRDS) supported by the National Institute of Diabetes and Digestive and Kidney Diseases (NIDDK), the incidence of CKD stage 5 in children has appeared to decline slightly, with 17.5 per million population (PMP) in 2004, declining by 21.7% to 13.8 PMP in 2015 [21]. Of those receiving kidney transplantation, 25% may require dialysis in the course of the disease [22,23]. Available data suggest that children are currently treated both by peritoneal and hemodialysis [24,25].

Compared to CVCs, AVFs are associated with lower infection rates, higher central vein patency rates, and higher quality during dialysis [10,26,27]. HD catheter-related complications are associated with up to 8-fold higher mortality compared to those resulting from AVFs [28,29,30,31]. Taking this into account, AVGs should be considered as an exception for HD access in children in whom the creation of native vessels is not possible to perform an adequate anastomosis [8]. The possibility of HD in children was first described in the 1960s. Fine et al. shared their experience of five adolescent patients awaiting transplantation using a silastic Teflon arterio-venous cannula and low-resistance pumpless no-prime hemodialyzer for 1–6 months for intermittent hemodialysis 2 to 3 times weekly [32]. This method had technical success without serious complications. The first surgically created AVF for the purpose of HD in adults was described by Brescia et al. in 1966 and subsequently became the preferred vascular access point, mainly due to its low complication rates and long life span [33]. Although Brescia-Cimino fistulas can be constructed with a high success rate in adults, numerous difficulties are encountered in establishing such access in children [34,35], including limitations by small vessel diameters, lower arterial flows, and potential vasospasms [28].

Several studies have described success for the creation of AVFs in pediatric populations with variable rates of primary failure in the range of 5.5–33.3% [36,37,38,39]. In our study, primary failure was 10.5%, and therefore comparable to previously published results. Despite these promising numbers, different risk factors have been described that tend to complicate vascular access creation in children. In 1973, Broyer et al. performed radial-cephalic AVFs in low-weight pediatric patients, achieving success rates as low as 54% in children with less than 20 kg bodyweight (BW) [40]. The first distal AVF using microsurgical techniques in children with less than 10 kg of BW was reported by Bourquelot et al. [41]. After the successful introduction of distal radial-cephalic AVFs, they presented excellent results of 434 access points in 380 children with an immediate patency of 96% and showed that distal AVFs created by microsurgery could successfully be used for HD access in pediatric patients [42]. While early predictors of fistula maturation were described in adults, similar data for children are scarce. Karava et al. analyzed 48 AVFs in a cohort of 41 children with less than 20 kg of BW, observing primary maturation in 29 subjects (60.4%) and 19 early complications. The median time to the first cannulation was 18.8 weeks (range 2–166.3). They found a significantly higher secondary patency rate in patients aged ≥ 3 years or weighing ≥ 13 kg at fistula creation [15].

Different fistula patency rates have been described between forearm and upper arm fistulas, as primary failure is more common in forearm fistulas [43]. According to a systematic review in order to assess outcomes of AVFs in elderly patients comparing both locations, forearm fistulas showed lower 1-year primary and secondary patency rates [44]. These data are comparable to the durability of AVFs in the pediatric population [12,15,17,39,45]. In our study, the primary patency rate was 67.6%, and the secondary patency rate was 87.3% after 1 year, with no difference between forearm and upper arm AVFs.

Several studies assessed additional factors to determine their potential influence on patency rates. Kim et al. evaluated 52 AVFs in 47 patients with cumulative 1-, 3-, and 5-year primary patency rates of 60.5%, 51.4%, and 47.7%, and secondary patency rates of 82.7%, 79.2%, and 79.2%, respectively [45]. They did not find any significant associations between patency rates, age, body weight, AVF type, the use of anticoagulation therapy, the presence of a central venous catheter, or history of vascular access failure. Primary failure was more frequently observed in patients with a low BW when it was evaluated as a continuous variable. Similarly, in our study, we did not find a significant association between patency rates and age, fistula localization, or time on hemodialysis before fistula creation. Normal BW seems to have a benefit in view of durability, although without proven significance in our data. A direct comparison of this study with the results previously published is not possible, as age-adjusted weight categories were used and not the continuous weight of the patients.

This study has several limitations. Firstly, only retrospectively collected data were available for this study. However, high value was placed on clean data collection, which is why general practitioners and dialysis centers were contacted in order to close gaps in our own records. Nevertheless, it was not possible to ensure a complete recording of all individual interventions or operations, as some of these were carried out at other centers. Secondly, the performance of each intervention was recorded, but not the exact procedure which was conducted. Therefore, the patency rates could be calculated correctly, but no detailed analysis of the secondary interventions could be carried out over such a long follow-up period. Thirdly, even though some of the patients had regular follow-up examinations, some of which included an ultrasound examination of the shunt, these data are only sporadically available and not continuously documented. It is, therefore, not possible to analyze the data, even if they are interesting, as these data support the data’s validity. Fourthly, further analysis of the hemoglobin values as well as the intraoperative anticoagulation regime was not possible due to the study’s retrospective character.

Despite these limitations, this study has one of the longest follow-ups available and is therefore well suited to provide a general picture of the longevity of an AVF in children. The primary failure rates, as well as the patency rates, are comparable to those reported in adults. The primary failure of 10.5% reported in the pediatric population lies in the normal range of that reported in adults, 5–46%, which is also the case for the patency rates (i.e., one-year secondary patency rate) [46]. Therefore, our findings further support the recommendations of current international guidelines preferring AVF to CVC in the pediatric patient population [22,47].

## 5. Conclusions

In our study cohort, AVFs showed an acceptable rate of primary failure and excellent long-term patency. In this context, AVFs are an appropriate option for dialysis access in pediatric patients.

## Figures and Tables

**Table 1:** Patients and procedure characteristics.

Age, Years (Median, Range)	15 (7–18)
Sex (*n*, %)	
Male	29 (51)
Female	28 (49)
Weight, kg (median, range)	47 (19–81)
Etiology of renal failure (*n*, %)	
Glomerulonephritis	25 (44)
Obstructive uropathy	23 (40)
Metabolic diseases	5 (9)
Metacystic dysplastic kidney	2 (4)
Unknown	2 (4)
Dialysis at time point of surgery (*n*, %)	17 (30)
Hemodialysis (via central venous catheter)	20 (35)
Peritoneal dialysis	4 (7)
Fistula configuration (*n*, %)	
**Forearm**	**54 (95)**
Radial artery/cephalic vein	51 (89)
Radial artery/basilic vein	2 (4)
Ulnar artery/basilic vein	1 (2)
**Upper arm**	**3 (5)**
Cephalic vein	2 (4)
Basilic vein	1 (2)

## Data Availability

Anonymized data can be requested from the authors.
